# In‐Vitro Activity of Ceftazidime‐Avibactam and Comparator Agents Against Carbapenem‐Resistant Gram‐Negative Isolates at a Tertiary Care Hospital in Lahore, Pakistan: A Cross‐Sectional Study

**DOI:** 10.1002/mbo3.70449

**Published:** 2026-11-03

**Authors:** Faryal Yunus, Obaid Ur Rehman, Muhammad Murtaza, Safa Mazhar, Abedin Samadi, Ahmed Asad Raza

**Affiliations:** ^1^ Department of Pathology (Microbiology) National Hospital and Medical Centre Lahore Punjab Pakistan; ^2^ Services Institute of Medical Sciences Lahore Punjab Pakistan; ^3^ Department of Medicine Jinnah Sindh Medical University Karachi Sindh Pakistan; ^4^ Department of Medicine Kabul University of Medical Sciences Abu Ali Sina Kabul Kābul Afghanistan

**Keywords:** antimicrobial resistance, carbapenem‐resistant Enterobacterales, Ceftazidime‐avibactam, Pakistan, β‐lactamases

## Abstract

Carbapenem‐resistant Gram‐negative bacteria are WHO critical priority pathogens with few treatment options. Ceftazidime‐avibactam (CAZ–AVI) inhibits serine carbapenemases (KPC, OXA‐48‐like) but not metallo‐β‐lactamases, which are common in South Asia. Local data are scarce; we assessed CAZ–AVI and comparator activity in Pakistan. In this laboratory‐based cross‐sectional study, consecutive non‐duplicate carbapenem‐resistant isolates from a tertiary care centre in Lahore (December 2024–May 2025) were tested by MIC and disc diffusion with CLSI interpretation. The primary outcome was CAZ–AVI susceptibility with exact binomial 95% confidence intervals; comparisons used Fisher's exact test, McNemar's test, and multivariable logistic regression. Of 210 isolates, 200 had CAZ–AVI results; susceptibility was 70.5% (141/200; 95% CI 63.7–76.7). It varied by sample source (*p* = 0.008), from 81.2% in blood cultures to 38.5% (5/13) in other specimens, but not by organism, department, age, or gender. In paired comparisons, CAZ–AVI susceptibility exceeded gentamicin, ciprofloxacin, piperacillin‐tazobactam, and trimethoprim‐sulfamethoxazole, was similar to amikacin, and was lower than colistin, fosfomycin, and tigecycline. Among 59 resistant isolates, colistin (94.5%), fosfomycin (92.9%), tigecycline (79.5%), and chloramphenicol (76.7%) were most active. Sample source was the only independent predictor (other specimens vs blood culture: adjusted OR 0.18, 95% CI 0.05–0.68; imprecise). CAZ–AVI was active in vitro against 70.5% of carbapenem‐resistant Gram‐negative isolates. Because nearly one‐third were resistant, these findings support surveillance and susceptibility‐guided rather than empirical use. Metallo‐β‐lactamase production is a plausible but unconfirmed explanation. Molecular characterization and evaluation of CAZ–AVI plus aztreonam are needed.

## Graphical abstract

Of 210 consecutive non‐duplicate carbapenem‐resistant Gram‐negative isolates collected at a tertiary care hospital in Lahore, Pakistan (December 2024–May 2025), 200 had a ceftazidime‐avibactam (CAZ–AVI) result; 70.5% were susceptible (141/200; 95% CI 63.7–76.7). Susceptibility varied by sample source (*p* = 0.008), being highest in blood cultures (81.2%), and did not differ by organism, department, age, or sex. In paired comparisons, CAZ–AVI was more active in vitro than gentamicin, ciprofloxacin, piperacillin‐tazobactam, and trimethoprim‐sulfamethoxazole, comparable to amikacin, and less active than colistin, fosfomycin, and tigecycline. Among the 59 CAZ–AVI‐resistant isolates, colistin (94.5%), fosfomycin (92.9%), tigecycline (79.5%), and chloramphenicol (76.7%) were most active. These findings support susceptibility‐guided rather than empirical CAZ–AVI use, alongside molecular characterization of resistance mechanisms.

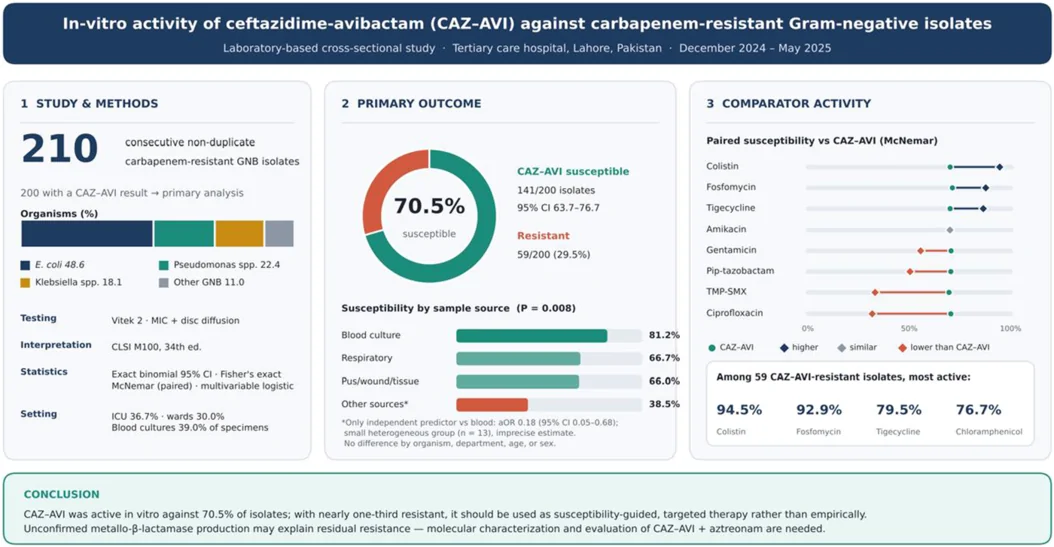

## 1 Introduction

Multidrug‐resistant (MDR) Gram‐negative bacteria are a serious public health threat all over the world. The misuse and overuse of broad‐spectrum antibiotics have enabled antimicrobial resistance to build up, making infections more difficult to treat. Among the most alarming resistant pathogens are carbapenem‐resistant Gram negative bacteria (CR‐GNB), which are linked to reduced treatment choices, higher health care expenditure, longer hospital stays, and a greater mortality risk (Xiao et al. 2023; Chen et al. 2022; Zhong et al. 2018).

Carbapenem‐resistant organisms are largely Enterobacterales, Pseudomonas aeruginosa, and Acinetobacter baumannii. They are known to cause severe infections like bloodstream infections, pneumonia, urinary tract infections, intra‐abdominal infections, and wound infections especially in the critically ill and immunocompromised patients. The primary mechanism of resistance is the production of carbapenemases such as Klebsiella pneumoniae carbapenemase (KPC), New Delhi metallo‐β‐lactamase (NDM) and oxacillinase‐48 (OXA‐48) that degrade carbapenems, significantly compromising their activity (Sternbach et al. 2018). The emergence of extensively drug‐resistant (XDR) and pan‐drug‐resistant (PDR) strains has added to the complexity of the clinical management and highlighted the need for effective therapeutic alternatives to address the increasing concern. To address this problem, the World Health Organization (WHO) has designated carbapenem‐resistant Enterobacterales (CRE), carbapenem‐resistant Pseudomonas aeruginosa (CRPA), and carbapenem‐resistant Acinetobacter baumannii (CRAB) as priority pathogens for new antimicrobial agents (Zhen et al. 2022).

Ceftazidime‐avibactam is an important new treatment for infections due to carbapenem‐resistant Gram‐negative bacteria, including CRE and Pseudomonas aeruginosa (Vena et al. 2020; Che et al. 2019). The combination is made up of ceftazidime, a third‐generation cephalosporin, and avibactam, a non‐β‐lactam β‐lactamase inhibitor. Avibactam restores the activity of ceftazidime by inhibiting class A (including KPC), class C (AmpC), and selected class D β‐lactamases such as OXA‐48 (Sharma et al. 2016). Ceftazidime‐avibactam has been shown to have a better safety profile compared with other older therapeutic agents, including colistin and tigecycline, with respect to nephrotoxicity (Zhong et al. 2018; Sharma et al. 2016). Clinical studies and systematic reviews have demonstrated that ceftazidime‐avibactam has led to improved clinical and microbiological outcomes as well as reduced mortality in patients with carbapenem‐resistant infections (Chen et al. 2022; Zhong et al. 2018; Sternbach et al. 2018). Resistance to ceftazidime‐avibactam has also been reported, especially when the isolate has a metallo‐β‐lactamase like NDM, and among some resistant strains of Klebsiella pneumoniae (Krithika et al. 2024). Therefore, continual monitoring of susceptibility patterns and resistance mechanisms is crucial to ensure its continued clinical usefulness.

The overuse of antibiotics, poor antimicrobial stewardship, and poor infection control practices are leading to antimicrobial resistance in Pakistan (Bilal et al. 2021). Tertiary care hospitals are particularly at risk of dissemination of CR‐GNB, because of the high proportion of critically ill patients and intensive exposure to antibiotics. Although ceftazidime‐avibactam has been used in clinical practice, there is still a lack of local data on the in‐vitro activity of ceftazidime‐avibactam against carbapenem‐resistant Gram‐negative isolates.

Hence, the objective of the present study was to assess the in‐vitro activity of ceftazidime‐avibactam and comparator agents against carbapenem resistant Gram‐negative isolates obtained from clinical specimens in a tertiary care hospital, Lahore, Pakistan. In addition, associations between patient characteristics, specimen sources and CAZ‐AVI susceptibility were evaluated. The results are intended to inform local antimicrobial surveillance and stewardship and to support susceptibility‐guided treatment decisions for MDR Gram‐negative infections.

## 2 Methods

### 2.1 Study Design and Isolates

We conducted a laboratory‐based, cross‐sectional analysis of consecutive non‐duplicate carbapenem‐resistant Gram‐negative bacterial isolates recovered from routine clinical specimens at National Hospital and Medical Center, Lahore, between December 2024 and May 2025. An isolate was classified as carbapenem‐resistant if it was non‐susceptible to meropenem or imipenem according to Clinical and Laboratory Standards Institute (CLSI) M100 performance standards (34th edition). For patients with multiple isolates, only the first non‐duplicate isolate per patient–organism combination was retained. For each isolate, we extracted patient age, gender, requesting department, specimen type, species identification, and the complete antibiotic susceptibility profile. Isolates were recovered from specimens submitted for routine diagnostic purposes. Because the laboratory dataset did not record whether an isolate represented infection or colonization, antibiotic exposure before specimen collection, or patient outcomes, the unit of analysis was the isolate rather than the clinical infection episode. Species identification and antimicrobial susceptibility testing were performed using the Vitek 2 automated system, with ceftazidime‐avibactam (CAZ–AVI) tested by Minimum Inhibitory Concentration (MIC) and Disc Diffusion Method; results were interpreted according to Clinical and Laboratory Standards Institute (CLSI) M100 performance standards (34th edition). The study was approved by the IRB/ETHICS COMMITTEE, REF:NHMC/IRB/155. No formal a priori sample‐size calculation was performed; all consecutive eligible isolates identified during the study period were included, representing a complete‐enumeration convenience sample. To limit bias, only the first non‐duplicate isolate per patient–organism combination was analyzed, species identification and CLSI‐based interpretation were applied uniformly to every isolate, and a pre‐specified sensitivity analysis assessed the influence of duplicate laboratory accession numbers. Reporting follows the STROBE statement for cross‐sectional studies.

### 2.2 Subgroup Categorization

For subgroup analysis, organism, sample source, and department variables were collapsed into clinically meaningful groups to reduce sparse‐cell instability. Organisms were categorized as *E. coli*, *Klebsiella* spp., *Pseudomonas* spp., and other Gram‐negative organisms with the last group including less frequent isolates such as *Serratia* spp., *Proteus* spp., *Enterobacter* spp., *Acinetobacter* spp., and *Morganella* spp. Sample sources were grouped as blood culture, respiratory samples, pus/wound/tissue/body fluid, and other sample sources, with the last category comprising rare or mixed specimen types including catheter tips, drain fluids, genital/HVS samples, and other uncommon specimens. Departments were grouped as ICU/critical care, HDU, inpatient ward, and OPD/outpatient. The “other” categories in each variable represent recorded but infrequent or heterogeneous categories, not missing values.

### 2.3 Statistical Analysis

Analyses were carried out at the isolate level in R (version 4.4). The primary outcome was the proportion of isolates susceptible to CAZ–AVI, reported with an exact binomial 95% confidence interval. Isolates without a CAZ–AVI result were excluded from the primary analysis. Among CAZ–AVI‐resistant isolates, susceptibility to each comparator antibiotic was calculated using the number of isolates actually tested for that drug as the denominator. Susceptibility of the full cohort to every tested comparator antibiotic was likewise summarized with drug‐specific denominators (number tested and number susceptible) and exact binomial 95% confidence intervals.

CAZ–AVI susceptibility was stratified by organism group, sample source, department, age group, and gender, and compared across categories using Fisher's exact test with simulated *p*‐values. A multivariable logistic regression model was fitted to identify variables independently associated with CAZ–AVI susceptibility, including organism group, sample source, department, and gender; age group was excluded from the model due to complete separation in the < 18 stratum (100% susceptibility). Results are reported as adjusted odds ratios (aOR) with 95% confidence intervals. To compare CAZ–AVI with each major comparator antibiotic on a paired basis, McNemar's test was applied to isolates tested for both agents.

To assess the impact of duplicate laboratory accession numbers, sensitivity analyses repeated the primary estimate after (i) retaining only the first record per duplicated lab number and (ii) excluding all duplicated records. Missing or not‐tested susceptibility results were handled by complete‐case analysis within each variable and antibiotic, with denominators restricted to isolates actually tested for the relevant drug; no imputation was performed. All figures were produced in ggplot2.

## 3 Results

### 3.1 Cohort and Overall CAZ–AVI Susceptibility

Of 210 carbapenem‐resistant Gram‐negative isolates, 200 had a CAZ–AVI result available and formed the primary analysis set. Patient and isolate characteristics are summarized in Table 1. The cohort was predominantly from older patients (77.6% aged > 40 years) and inpatient settings, mainly ICU/critical care (36.7%) and inpatient wards (30.0%). Blood cultures contributed the largest share of specimens (39.0%), and *E. coli* was the most frequent organism (48.6%), followed by *Pseudomonas* spp. (22.4%), *Klebsiella* spp. (18.1%), and other Gram‐negative species (11.0%).

**Table 1:** Demographic, clinical‐source, and microbiological characteristics of carbapenem‐resistant Gram‐negative isolates according to ceftazidime‐avibactam susceptibility.

Variable	Category	All isolates, *n* (%)	CZA‐tested isolates, *n*	CZA susceptible, *n*/*N* (%; 95% CI)	CZA resistant, *n*/*N* (%)	*p* value
Overall	All CZA‐tested isolates	210 (100.0)	200	141/200 (70.5%; 95% CI 63.7–76.7)	59/200 (29.5%)	
Organism group	*E. coli*		95	70/95 (73.7%; 95% CI 63.6–82.2)	25/95 (26.3%)	0.7889
	*Klebsiella spp*.		35	23/35 (65.7%; 95% CI 47.8–80.9)	12/35 (34.3%)	
	*Pseudomonas spp*.		47	32/47 (68.1%; 95% CI 52.9–80.9)	15/47 (31.9%)	
	Other Gram‐negative organisms		23	16/23 (69.6%; 95% CI 47.1–86.8)	7/23 (30.4%)	
Sample source	Blood culture	82 (39.0)	80	65/80 (81.2%; 95% CI 71.0–89.1)	15/80 (18.8%)	0.0077
	Respiratory	60 (28.6)	54	36/54 (66.7%; 95% CI 52.5–78.9)	18/54 (33.3%)	
	Pus/wound/tissue/body fluid	54 (25.7)	53	35/53 (66.0%; 95% CI 51.7–78.5)	18/53 (34.0%)	
	Other sample source	14 (6.7)	13	5/13 (38.5%; 95% CI 13.9–68.4)	8/13 (61.5%)	
Department	ICU/critical care	77 (36.7)	71	54/71 (76.1%; 95% CI 64.5–85.4)	17/71 (23.9%)	0.3418
	Inpatient ward	63 (30.0)	62	45/62 (72.6%; 95% CI 59.8–83.1)	17/62 (27.4%)	
	HDU	34 (16.2)	33	20/33 (60.6%; 95% CI 42.1–77.1)	13/33 (39.4%)	
	OPD/outpatient	36 (17.1)	34	22/34 (64.7%; 95% CI 46.5–80.3)	12/34 (35.3%)	
Age group	< 18	10 (4.8)	10	10/10 (100%; 95% CI 69.2–100)	0/10 (0.0%)	0.1350
	18‐40	37 (17.6)	34	24/34 (70.6%; 95% CI 52.5–84.9)	10/34 (29.4%)	
	41‐60	66 (31.4)	63	41/63 (65.1%; 95% CI 52.0–76.7)	22/63 (34.9%)	
	> 60	97 (46.2)	93	66/93 (71.0%; 95% CI 60.6–79.9)	27/93 (29.0%)	
Gender	Male	108 (51.4)	101	68/101 (67.3%; 95% CI 57.3–76.3)	33/101 (32.7%)	0.3543
	Female	102 (48.6)	99	73/99 (73.7%; 95% CI 63.9–82.1)	26/99 (26.3%)	

*Note: p*‐values compare susceptibility across each full subgroup using Fisher's exact test. “Other” groups include rare or mixed recorded categories. Department records previously classified as other/unclear were reassigned to ICU/critical care and inpatient ward before final analysis.

Abbreviations: CZA, ceftazidime‐avibactam; CI, confidence interval; ICU, intensive care unit; HDU, high‐dependency unit; OPD, outpatient department.

CAZ–AVI retained activity against 70.5% of isolates (141/200; 95% CI 63.7–76.7) (Figure 1). Across the broader susceptibility panel (Table 3), only colistin (94.8%, 184/194), fosfomycin (87.1%, 135/155), tigecycline (86.6%, 136/157), and chloramphenicol (82.4%, 131/159) showed higher in‐vitro susceptibility than CAZ–AVI, although the number of isolates tested differed between agents; nitrofurantoin was susceptible in 10/11 tested isolates (90.9%), although the small denominator limits interpretation. All other β‐lactams and β‐lactam/β‐lactamase inhibitor combinations showed substantially lower susceptibility (Table 3; Supporting Information S1: Figure 1).

**Figure 1:**
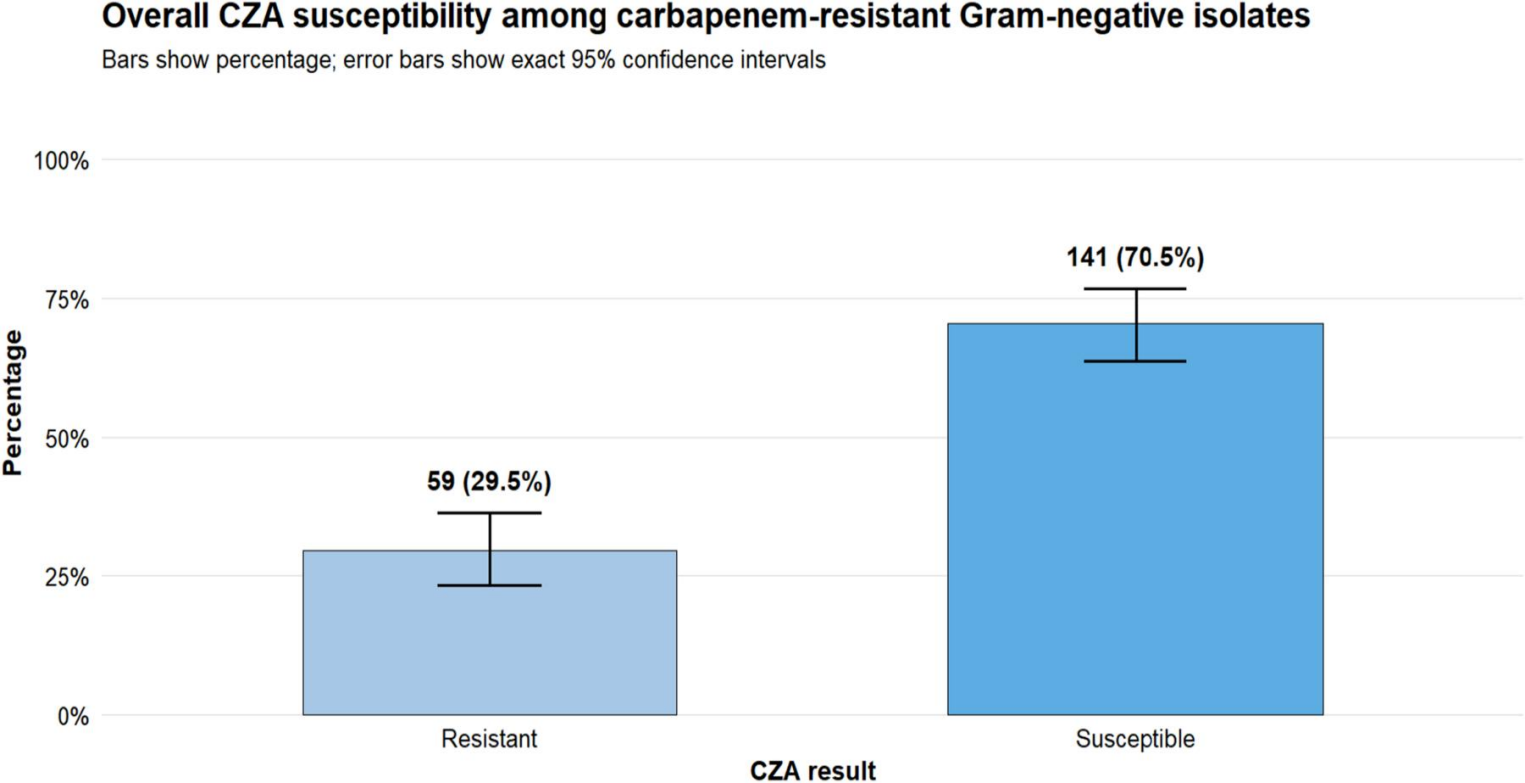
Overall ceftazidime‐avibactam susceptibility and resistance among carbapenem‐resistant Gram‐negative isolates, with 95% confidence intervals.

### 3.2 Subgroup Analyses

CAZ–AVI susceptibility was broadly similar across organism groups, ranging from 65.7% in *Klebsiella* spp. to 73.7% in *E. coli* (*p* = 0.80), and across departments, ranging from 60.6% in HDU (20/33) to 76.1% in ICU/critical care (54/71), with no significant difference across department groups (*p* = 0.3418) (Supporting Figures 2 and 3). Susceptibility differed significantly by sample source (*p* = 0.008): blood‐culture isolates had the highest susceptibility (81.2%, 65/80), whereas the small heterogeneous “other sample source” group had the lowest (38.5%, 5/13; 95% CI 13.9–68.4); respiratory and pus/wound isolates fell between these (66.7% and 66.0%, respectively) (Supporting Figure 4). Because this group was small and comprised clinically heterogeneous specimen types, the estimate is imprecise and should be interpreted with caution. No significant differences were observed by age group (*p* = 0.13) or gender (73.7% in females vs 67.3% in males; *p* = 0.35) (Supporting Information S1: Figures 5 and 6).

The organism antibiotic susceptibility matrix (Figure 2) showed the expected patterns: colistin and tigecycline showed high in‐vitro activity against the Enterobacterales, fosfomycin was most active against *E. coli*, amikacin outperformed gentamicin overall, and piperacillin‐tazobactam, trimethoprim‐sulfamethoxazole, the fluoroquinolones, and the carbapenems showed limited or no activity across organism groups.

**Figure 2:**
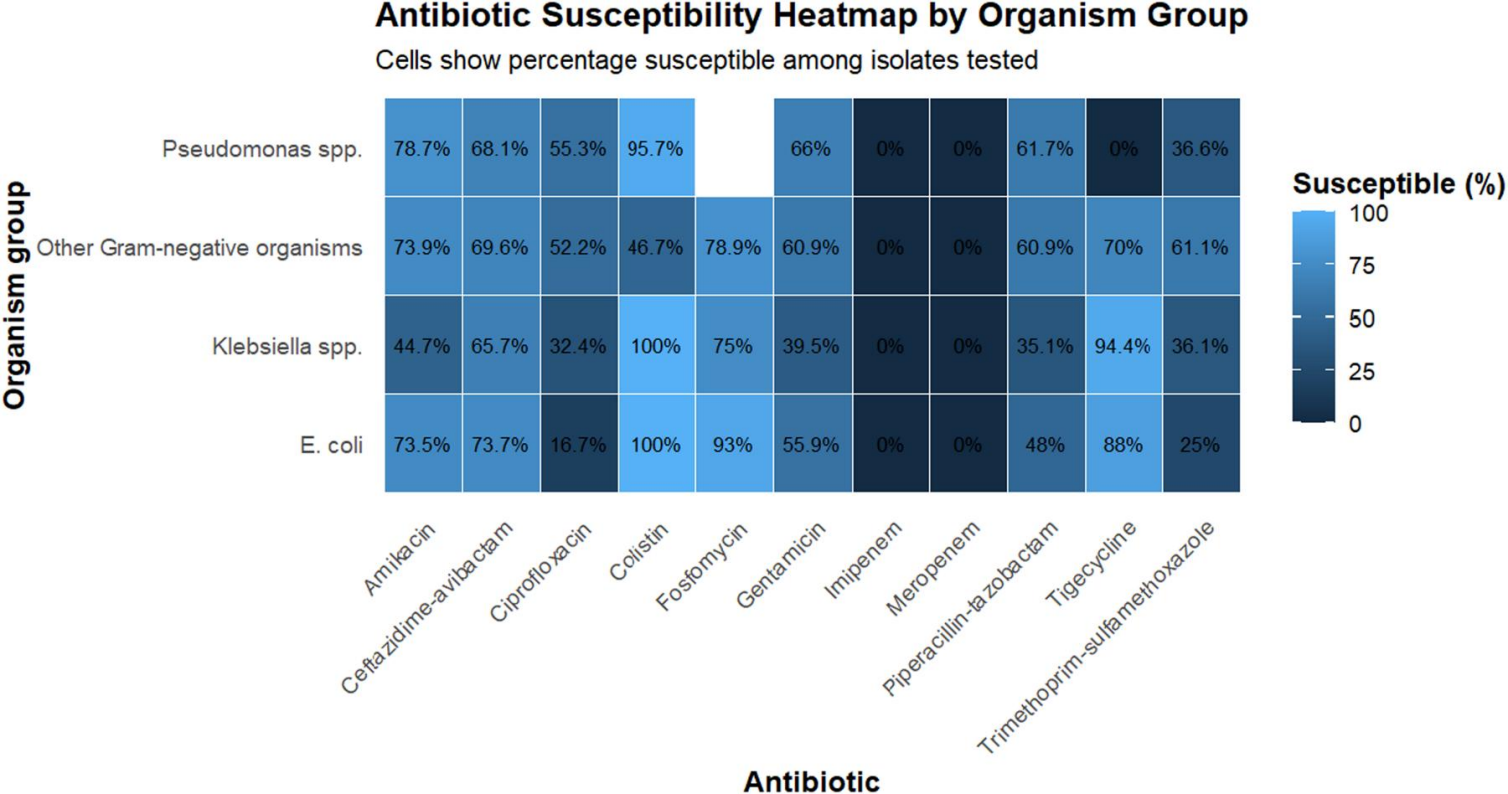
Heatmap of organism‐wise susceptibility percentages for ceftazidime‐avibactam and comparator antibiotics among carbapenem‐resistant Gram‐negative isolates.

### 3.3 Activity of Alternative Antibiotics Among CAZ–AVI‐Resistant Isolates

Among the 59 CAZ–AVI‐resistant isolates, in‐vitro co‐susceptibility to alternative antibiotics is summarized in (Table 2). The highest susceptibility was observed for colistin (94.5%, 52/55), fosfomycin (92.9%, 39/42), tigecycline (79.5%, 35/44), and chloramphenicol (76.7%, 33/43); nitrofurantoin was susceptible in all three tested isolates, although the small denominator limits interpretation. Activity of conventional Gram‐negative agents was substantially reduced. Amikacin retained 42.4% susceptibility (25/59) and outperformed gentamicin (32.2%, 19/59) and tobramycin (20.0%, 4/20); doxycycline (38.6%, 17/44) and trimethoprim‐sulfamethoxazole (27.3%, 15/55) showed modest activity. Fluoroquinolones were largely inactive (ciprofloxacin and levofloxacin, both 11.9%, 7/59 each), as were piperacillin‐tazobactam (10.2%, 6/59), cefepime (7.4%, 4/54), ceftazidime (6.2%, 1/16), and amoxicillin‐clavulanate (2.3%, 1/43). No CAZ–AVI‐resistant isolate was susceptible to ampicillin, cefixime, ceftriaxone, cefuroxime, meropenem, or imipenem (0/43 to 0/59 per drug). Exact 95% confidence intervals for each agent are shown in (Supporting Figure 7).

**Table 2:** In‐vitro co‐susceptibility to alternative antibiotics among ceftazidime‐avibactam‐resistant isolates.

Antibiotic	CZA‐resistant isolates tested, *n*	Susceptible, *n*/*N* (%; 95% CI)	Non‐susceptible, *n*/*N* (%)
Colistin	55	52/55 (94.5%; 95% CI 84.9–98.9)	3/55 (5.5%)
Fosfomycin	42	39/42 (92.9%; 95% CI 80.5–98.5)	3/42 (7.1%)
Tigecycline	44	35/44 (79.5%; 95% CI 64.7–90.2)	9/44 (20.5%)
Chloramphenicol	43	33/43 (76.7%; 95% CI 61.4–88.2)	10/43 (23.3%)
Amikacin	59	25/59 (42.4%; 95% CI 29.6–55.9)	34/59 (57.6%)
Doxycycline	44	17/44 (38.6%; 95% CI 24.4–54.5)	27/44 (61.4%)
Gentamicin	59	19/59 (32.2%; 95% CI 20.6–45.6)	40/59 (67.8%)
Trimethoprim‐sulfamethoxazole	55	15/55 (27.3%; 95% CI 16.1–41.0)	40/55 (72.7%)
Ciprofloxacin	59	7/59 (11.9%; 95% CI 4.9–22.9)	52/59 (88.1%)
Levofloxacin	59	7/59 (11.9%; 95% CI 4.9–22.9)	52/59 (88.1%)
Piperacillin‐tazobactam	59	6/59 (10.2%; 95% CI 3.8–20.8)	53/59 (89.8%)
Cefepime	54	4/54 (7.4%; 95% CI 2.1–17.9)	50/54 (92.6%)

*Note:* Susceptibility is reported as n/N (%; 95% CI), where N is the number of CZA‐resistant isolates tested for each antibiotic. Non‐susceptible includes intermediate and resistant results. Missing or not‐tested results were excluded from each antibiotic‐specific denominator. Results represent in‐vitro co‐susceptibility patterns only, not clinical treatment superiority.

In paired comparisons restricted to isolates tested for both CAZ–AVI and the comparator (Supporting Information S1: Figure 8), CAZ–AVI showed significantly higher susceptibility than gentamicin (70.5% vs 55.5%; *p* < 0.001), ciprofloxacin (70.4% vs 31.7%; *p* < 0.001), piperacillin‐tazobactam (70.4% vs 50.3%; *p* < 0.001), and trimethoprim‐sulfamethoxazole (69.6% vs 33.1%; *p* < 0.001) on McNemar's test. CAZ–AVI and amikacin were comparable (70.5% vs 70.0%; *P* = 1.00). Conversely, colistin (94.6% vs 70.1%; *p* < 0.001), tigecycline (86.4% vs 70.1%; *p* < 0.001), and fosfomycin (87.7% vs 71.2%; *p* = 0.002) all showed significantly higher susceptibility than CAZ–AVI in the paired analysis. These paired comparisons describe differences in in‐vitro susceptibility only and do not indicate differences in clinical effectiveness.

### 3.4 Independent Predictors of CAZ–AVI Susceptibility

In multivariable logistic regression, sample source was the only variable independently associated with CAZ–AVI susceptibility. Compared with blood‐culture isolates, isolates from other sample sources had significantly lower odds of CAZ–AVI susceptibility (aOR 0.18, 95% CI 0.05–0.68; *p* = 0.013). However, this category comprised only 13 CAZ–AVI‐tested isolates, and the wide confidence interval indicates considerable imprecision. Respiratory samples (aOR 0.52, 95% CI 0.21–1.28; *p* = 0.158) and pus/wound/tissue/body fluid samples (aOR 0.52, 95% CI 0.21–1.26; *p* = 0.149) showed numerically lower odds of susceptibility but did not reach statistical significance. Organism group, department, and gender were not independently associated with CAZ–AVI susceptibility. Using HDU as the reference, department‐level aORs were 1.60 for ICU/critical care, 1.68 for inpatient ward, and 1.20 for OPD/outpatient, with all confidence intervals crossing 1. Female gender showed no significant association compared with male gender (aOR 1.18, 95% CI 0.61–2.28; *p* = 0.633).

### 3.5 Sensitivity Analysis

The primary estimate was robust to handling of duplicate laboratory accession numbers: susceptibility was 71.7% (137/191; 95% CI 64.8–78.0) after keeping only the first record per duplicate, and 72.4% (131/181; 95% CI 65.3–78.7) after excluding all duplicates, both within the 95% CI of the primary estimate.

## 4 Discussion

### 4.1 Summary of Principal Findings

In this single‐center cohort, CAZ‐AVI showed in‐vitro activity against 70.5% of 200 carbapenem‐resistant Gram‐negative isolates primarily composed of *E. coli*, Pseudomonas spp., and Klebsiella spp., from inpatient settings. In paired in‐vitro comparisons, susceptibility to CAZ‐AVI was significantly higher than to piperacillin‐tazobactam, gentamicin, ciprofloxacin, and trimethoprim‐sulfamethoxazole, and significantly lower than to colistin, fosfomycin, and tigecycline. CAZ‐AVI susceptibility did not differ significantly across different bacterial species, patient demographics, and hospital wards. Multivariable logistic regression showed that sample source was the sole independent predictor of susceptibility, with blood culture isolates demonstrating the highest susceptibility (81.2%), whereas a small heterogeneous group of other specimen types (*n* = 13) had lower odds of susceptibility, an imprecise estimate that should not be overinterpreted. Because these data describe in‐vitro susceptibility, they do not establish the clinical efficacy of CAZ‐AVI or its superiority over comparator agents. With nearly one‐third of isolates resistant, they support local surveillance and targeted, susceptibility‐guided use of CAZ‐AVI rather than empirical use, particularly in severe infections.

### 4.2 Comparison With Existing Literature

Ceftazidime–avibactam (CAZ‐AVI) is a novel β‐lactam/β‐lactamase inhibitor combination that restores activity against many MDR Gram‐negative organisms by inhibiting class A (including KPC), class C (AmpC), and selected class D (notably OXA‐48‐like) β‐lactamases without activity against metallo‐β‐lactamases (MBLs) such as NDM and VIM producers. This mechanism restriction is especially significant in South Asian countries where NDM‐type carbapenemases are very common.

The present study showed that CAZ‐AVI exhibited an overall susceptibility rate of 70.5% in carbapenem‐resistant Gram‐negative bacteria (CR‐GNB), indicating substantial, but incomplete, in‐vitro activity in a high‐resistance hospital setting. International evidence published indicates that CAZ‐AVI activity is highly variable and closely associated with underlying carbapenemase epidemiology and not organism alone. In the very specific context of appropriate use, CAZ‐AVI has been shown to provide good clinical and microbiological results in CRE infections, especially those with KPC production, justifying its targeted use in these infections (Shields et al. 2016). Likewise, Tumbarello et al. reported better survival rates for patients receiving CAZ‐AVI as compared to older regimens in cases of bloodstream infection with KPC‐producing Klebsiella pneumoniae (Tumbarello et al. 2021).

However, resistance is much more likely to be present in areas where MBLs are predominant, because avibactam does not inhibit these enzymes (van Duin and Bonomo 2016). This is consistent with surveillance data showing reduced in‐vitro susceptibility in South Asia and some parts of the Middle East where both NDM and OXA‐48‐like enzymes are present (van Duin and Bonomo 2016; Logan and Weinstein 2017).

In a global perspective, our results fit the WHO classification of CRE, Pseudomonas aeruginosa and Acinetobacter baumannii as “Critical Priority Pathogens,” reinforcing the need for new antimicrobial agents and stewardship optimization (Organization 2024). In this context, a ~70% susceptibility rate indicates that CAZ‐AVI retains in‐vitro activity against most, but not all, isolates; its use should therefore be guided by susceptibility testing and, where available, rapid detection of resistance mechanisms, rather than by empirical selection.

A large multicenter study reported antimicrobial resistance patterns in 11,185 gram‐negative isolates collected from hospitalized patients in US medical centers who were diagnosed with pneumonia and their results displayed CAZ‐AVI was successful in inhibiting 99.4% of *Klebsiella* spp. isolates with an extended‐spectrum B‐lactamase (ESBL) phenotype, whereas the susceptibility rate of these isolates to meropenem was only 64.7% (Sader et al. 2017 Jan 10). Furthermore, CAZ‐AVI was susceptible against 99.8% of *Enterobacter* spp. isolates compared to meropenem which was susceptible against 92.9% of the isolates (Sader et al. 2017 Jan 10). 99.9% of all *Enterobacteriaceae* species were inhibited by CAZ‐AVI at a dose of < 8 μg/mland among the 3402 *P. aeruginosa* isolates, the susceptibility rate for CAZ–AVI was 96.6% (Sader et al. 2017 Jan 10). These data reflect high in‐vitro activity in a setting where MBLs were uncommon and are therefore not directly comparable with our cohort. For MBL‐producing organisms, a distinct therapeutic approach is required: the Infectious Diseases Society of America recommends the combination of CAZ‐AVI and aztreonam against Metallo‐β‐lactamase (MBL)‐producing gram negative bacterial infections (Tamma et al. 2024 Aug 7), and findings reported by Sempere and colleagues showed synergistic activity in sixteen out of 19 isolates (Sempere et al. 2022 Sep 14), whereas, Falcone et al. reported a lower 30‐day mortality rate in the group treated with CAZ‐AVI‐aztreonam combination as compared to the group treated with other active antibiotics (Falcone et al. 2020 May 12). This combination should be regarded as a strategy separate from CAZ‐AVI monotherapy, which is not expected to be active against MBL producers; its potential role in our setting could not be assessed because combination testing and molecular characterization were not performed.

Rubab and colleagues reported a susceptibility rate of 45.60% for CAZ‐AVI in CRPA specimens collected at a tertiary care center in Pakistan (Rubab et al. 2026 Apr. 30). Ahsan et al. reported in‐vitro susceptibility of CAZ‐AVI against CR‐GNB isolates from a hospital in Pakistan (Ahsan et al. 2025), and they reported a susceptibility rate of 11% (17 out of 158 isolates from various clinical samples), indicating lower in‐vitro susceptibility. Another study in Pakistani settings conducted by Mehwish et al. reported similar resistance patterns where CAZ‐AVI was resistant in 80.8% of *E. coli* isolates and 72.1% of *Klebsiella pneumoniae* isolates (Mehwish and Iftikhar 2023). The higher CAZ‐AVI resistance rates reported in Pakistan have been attributed to the increased prevalence of NDM‐producing *Enterobacteriaceae* (Mehdi et al. 2025 Feb 21). These results indicate a need for judicious use of CAZ‐AVI as monotherapy in patients with sepsis where the use should be preceded by checking resistance to CAZ‐AVI (Mehdi et al. 2025 Feb 21). The higher susceptibility observed in our cohort than in some previous Pakistani reports may reflect differences in organism mix, specimen types, patient populations, testing methods, and local carbapenemase distribution, which we could not assess directly.

### 4.3 Possible Explanations for the Resistance Patterns

A plausible determinant is the local distribution of carbapenemase enzymes. CAZ‐AVI is generally active in vitro against KPC and OXA‐48‐like β‐lactamases, but is not active against MBLs (including NDM, VIM, and IMP types) (Zhong et al. 2018). In areas with a high prevalence of NDM‐producing Enterobacteriaceae such as South Asia, reduced susceptibility may be observed depending on the local prevalence of MBL‐producing strains (Zhong et al. 2018; Sternbach et al. 2018). However, because carbapenemase genes were not characterized in this study, the contribution of MBLs to the resistance observed in our cohort remains a hypothesis consistent with regional epidemiology rather than an established explanation. Other mechanisms may also contribute, including KPC variants with altered avibactam interactions, porin loss, efflux pump overexpression, and AmpC hyperproduction, the last being particularly relevant for *Pseudomonas* spp (Krithika et al. 2024; van Duin and Bonomo 2016).

Furthermore, gaps in antibiotic stewardship and pressure to prescribe antibiotics play a major role. A large number of MDR organisms emerge due to the high selective pressure of the spread of extensive use of broad‐spectrum antibiotics in tertiary care centers. In hospitals, poor use of antimicrobials has been associated with the spread and emergence of resistant clones (Prestinaci et al. 2015). This is especially important in high‐burden areas where antibiotic regulation and antibiotic de‐escalation may not be standard.

Other infection control measures also affect the spread of resistance. Nosocomial spread of resistant organisms is promoted by overcrowding in wards, frequent patient turnover, and inconsistent compliance with hand hygiene and contact precautions. Antibiotic use and invasive procedures are common in intensive care units (ICUs), which helps these conditions lead to the clonal expansion of carbapenem‐resistant strains, especially in ICUs (Prestinaci et al. 2015).

The variation observed across specimen types may also have biological explanations, although the difference was driven largely by a small heterogeneous group and should not be overinterpreted. The higher susceptibility of bloodstream isolates may be due to their earlier detection and less established biofilm formation than respiratory and wound isolates. However, respiratory tract infections and wound infections are frequently linked with long hospital stays, mechanical ventilation, and the presence of indwelling devices that create conditions conducive to the development of biofilm and enhance the development of antibiotic resistance phenotypes (Hall‐Stoodley et al. 2004).

In general, the susceptibility profile seen in this study probably reflects a combination of molecular resistance mechanisms, antimicrobial selection pressure, and healthcare‐associated transmission environment; however, these contributions could not be quantified in this laboratory‐based study. This highlights the need for the integration of molecular diagnostics, antimicrobial stewardship and infection prevention strategies to ensure the clinical utility of CAZ‐AVI.

### 4.4 Limitations and Future Directions

Despite the findings presented in our study, there are several key limitations that should be taken into account while interpreting the results. Firstly, the single‐center design and relatively short (6‐month) study period of our study might limit generalizability of the results which might vary from other patient populations and hospitals having different antibiotic use practices. Secondly, carbapenemase genes (KPC, OXA‐48‐like, NDM, VIM, and IMP) were not characterized, so the mechanisms underlying CAZ‐AVI resistance could not be determined and our mechanistic interpretations remain hypothetical.

Thirdly, the study measured in‐vitro susceptibility only; clinical response, microbiological eradication, treatment failure, and mortality were not assessed, and the results cannot be used to infer the clinical efficacy or superiority of any agent. Fourthly, the analysis was performed at the isolate level on specimens collected for routine diagnostic purposes. Information distinguishing infection from colonization, and on antibiotic exposure before specimen collection, was not available; susceptibility proportions therefore cannot be extrapolated directly to the proportion of treatable infections in the hospital population. The importance of clinical context is illustrated by device‐associated infections, in which management depends on microbiological assessment, individualized antibiotic therapy, and procedural factors, including definitive device management and local preventive strategies, rather than on antimicrobial susceptibility alone (Pandozi et al. 2024; Matteucci et al. 2023). Fifthly, the cohort combined clinically heterogeneous organisms and specimen types, and several subgroups, particularly the “other sample source” group (*n* = 13), were small, yielding imprecise estimates with wide confidence intervals. Finally, the number of isolates tested varied considerably between antibiotics because not every agent was tested against every isolate in routine practice; for example, fosfomycin, tigecycline, and gentamicin were tested in 42, 44, and 59 CAZ‐AVI‐resistant isolates, respectively (Table 2), and denominators in the full cohort ranged from 11 to 210 (Table 3). Comparisons between antibiotics may therefore be affected by these differing denominators, and the apparently high activity of agents tested in few isolates, such as nitrofurantoin, should not be overinterpreted.

**Table 3:** Susceptibility of all carbapenem‐resistant Gram‐negative isolates to ceftazidime‐avibactam and comparator antibiotics (full cohort, *n* = 210).

Antibiotic	Isolates tested, *N*	Susceptible, *n*/*N* (%; 95% CI)	Non‐susceptible, *n*/*N* (%)
Colistin	194	184/194 (94.8%; 95% CI 90.7–97.5)	10/194 (5.2%)
Nitrofurantoin	11	10/11 (90.9%; 95% CI 58.7–99.8)	1/11 (9.1%)
Fosfomycin	155	135/155 (87.1%; 95% CI 80.8–91.9)	20/155 (12.9%)
Tigecycline	157	136/157 (86.6%; 95% CI 80.3–91.5)	21/157 (13.4%)
Chloramphenicol	159	131/159 (82.4%; 95% CI 75.6–88.0)	28/159 (17.6%)
Ceftazidime‐avibactam	200	141/200 (70.5%; 95% CI 63.7–76.7)	59/200 (29.5%)
Amikacin	210	146/210 (69.5%; 95% CI 62.8–75.7)	64/210 (30.5%)
Azithromycin	18	12/18 (66.7%; 95% CI 41.0–86.7)	6/18 (33.3%)
Aztreonam	41	25/41 (61.0%; 95% CI 44.5–75.8)	16/41 (39.0%)
Gentamicin	210	117/210 (55.7%; 95% CI 48.7–62.5)	93/210 (44.3%)
Piperacillin‐tazobactam	209	105/209 (50.2%; 95% CI 43.3–57.2)	104/209 (49.8%)
Tobramycin	81	38/81 (46.9%; 95% CI 35.7–58.3)	43/81 (53.1%)
Doxycycline	158	74/158 (46.8%; 95% CI 38.9–54.9)	84/158 (53.2%)
Ceftazidime	50	18/50 (36.0%; 95% CI 22.9–50.8)	32/50 (64.0%)
Cefepime	187	66/187 (35.3%; 95% CI 28.5–42.6)	121/187 (64.7%)
Trimethoprim‐sulfamethoxazole	191	63/191 (33.0%; 95% CI 26.4–40.1)	128/191 (67.0%)
Ciprofloxacin	209	67/209 (32.1%; 95% CI 25.8–38.8)	142/209 (67.9%)
Levofloxacin	209	67/209 (32.1%; 95% CI 25.8–38.8)	142/209 (67.9%)
Amoxicillin‐clavulanate	160	40/160 (25.0%; 95% CI 18.5–32.4)	120/160 (75.0%)
Cefixime	161	34/161 (21.1%; 95% CI 15.1–28.2)	127/161 (78.9%)
Ceftriaxone	162	34/162 (21.0%; 95% CI 15.0–28.1)	128/162 (79.0%)
Cefuroxime	161	32/161 (19.9%; 95% CI 14.0–26.9)	129/161 (80.1%)
Ampicillin	159	7/159 (4.4%; 95% CI 1.8–8.9)	152/159 (95.6%)
Imipenem^a^	210	0/210 (0.0%; 95% CI 0.0–1.7)	210/210 (100.0%)
Meropenem^a^	210	0/210 (0.0%; 95% CI 0.0–1.7)	210/210 (100.0%)

*Note:* Susceptibility is reported as n/N (%; exact Clopper‐Pearson 95% CI), where N is the number of isolates in the full cohort tested for each antibiotic. Denominators vary because not every agent was tested against every isolate in routine practice; estimates based on small denominators (e.g., nitrofurantoin, azithromycin, aztreonam, ceftazidime) are imprecise and should be interpreted with caution. Non‐susceptible includes intermediate and resistant results. Missing or not‐tested results were excluded from each antibiotic‐specific denominator. ^a^All isolates were non‐susceptible to imipenem and meropenem by definition of the study population. Results represent in‐vitro susceptibility only and do not indicate clinical efficacy or treatment superiority.

Future research should focus on conducting multicenter large scale clinical trials and observational studies to establish the clinical effectiveness of CAZ‐AVI, together with molecular surveillance of carbapenemase genes and evaluation of CAZ‐AVI plus aztreonam against confirmed MBL producers. Furthermore, the research should take into account potential confounding factors such as age, gender, race, comorbidities, and prior exposure to antibiotics which can affect clinical outcomes.

## 5 Conclusion

CAZ‐AVI demonstrated in‐vitro activity against 70.5% of carbapenem‐resistant Gram‐negative isolates in this single‐center cohort. Because nearly one‐third of isolates were resistant, these findings do not support empirical CAZ‐AVI monotherapy in the absence of susceptibility data; rather, they support ongoing local antimicrobial surveillance and targeted CAZ‐AVI therapy guided by susceptibility testing, particularly in severe infections. Routine CAZ‐AVI susceptibility testing and molecular characterization of carbapenemase genes should be implemented to clarify resistance mechanisms, inform local treatment guidance, and mitigate further resistance.

## Author Contributions

**Faryal Yunus:** conceptualization, project administration, writing – original draft, writing – review and editing, supervision, investigation, data curation. **Obaid Ur Rehman:** writing – original draft, writing – review and editing, visualization, funding acquisition. **Muhammad Murtaza:** writing – original draft, writing – review and editing, resources. **Safa Mazhar:** writing – original draft, writing – review and editing. **Abedin Samadi:** writing – original draft, writing – review and editing. **Ahmed Asad Raza:** supervision, writing – original draft, writing – review and editing.

## Funding

The authors have nothing to report.

## Ethics Statement

This study was carried out after receiving approval from the Institutional Review Board of National Hospital & Medical Center, Lahore (NHMC/IRB/155; approved 19 June 2025). The requirement for informed consent was waived as all data were de‐identified before analysis.

## Conflicts of Interest

The authors declare no conflicts of interest.

## Supporting information


Supporting File

## Data Availability

The data that supports the findings of this study are available in the supporting material of this article. All data generated during the course of this study are included in this article.
