# Supplementary material for: In‐Vitro Activity of Ceftazidime‐Avibactam and Comparator Agents Against Carbapenem‐Resistant Gram‐Negative Isolates at a Tertiary Care Hospital in Lahore, Pakistan: A Cross‐Sectional Study
Source: Microbiologyopen. 2026 Nov 3;15(6):e70449. doi: 10.1002/mbo3.70449 (PMC13729263; doi:10.1002/mbo3.70449)
Supplement: Supplementary file 1 [file MBO3-15-e70449-s001.docx]

**Supplementary Material**

*In-vitro Activity of Ceftazidime-avibactam and Comparator Agents Against Carbapenem-resistant Gram-negative Isolates at a Tertiary Care Hospital in Lahore, Pakistan: a Cross-sectional Study*

**Supplementary Figure 1.** Overall antimicrobial susceptibility percentages for all tested antibiotics among carbapenem-resistant Gram-negative isolates, with 95% confidence intervals.

**Supplementary Figure 2.** Ceftazidime-avibactam susceptibility across organism groups, with 95% confidence intervals.

**Supplementary Figure 3.** Ceftazidime-avibactam susceptibility across final department categories, with 95% confidence intervals.

**Supplementary Figure 4.** Ceftazidime-avibactam susceptibility across specimen-source groups, with 95% confidence intervals.

**Supplementary Figure 5.** Ceftazidime-avibactam susceptibility across age groups, with 95% confidence intervals.

**Supplementary Figure 6.** Ceftazidime-avibactam susceptibility by gender, with 95% confidence intervals.

**Supplementary Figure 7.** Susceptibility to alternative antibiotics among ceftazidime-avibactam-resistant isolates, with 95% confidence intervals.

**Supplementary Figure 8.** Paired susceptibility comparisons between ceftazidime-avibactam and selected comparator antibiotics.
